# Supplementary material for: Ribonuclease 4 functions as an intestinal antimicrobial protein to maintain gut microbiota and metabolite homeostasis
Source: Nat Commun. 2024 Jul 10;15:5778. doi: 10.1038/s41467-024-50223-1 (PMC11237007; doi:10.1038/s41467-024-50223-1)
Supplement: Supplementary file 1 — Supplementary Information [file 41467_2024_50223_MOESM1_ESM.pdf]

Ribonuclease 4 functions as an intestinal antimicrobial protein to maintain gut microbiota and metabolite homeostasis

Jun Sun[#], Muxiong Chen[#], Zhen Hu[#], Ningqin Xu[#], Wenguang Wang, Zejun Ping, Jiayi Zhu, Desen Sun, Zhehao Zhu, Hangyu Li, Xiaolong Ge, Liang Luo, Wei Zhou, Rongpan Bai[*], Zhengping Xu[*], Jinghao Sheng[*]

#These authors contributed equally: Jun Sun, Muxiong Chen, Zhen Hu, Ningqin Xu

*These authors jointly supervised this work:

Jinghao Sheng (hsheng@zju.edu.cn)

Zhengping Xu (zpxu@zju.edu.cn)

Rongpan Bai (rpbai@zju.edu.cn)

Supplementary Figures

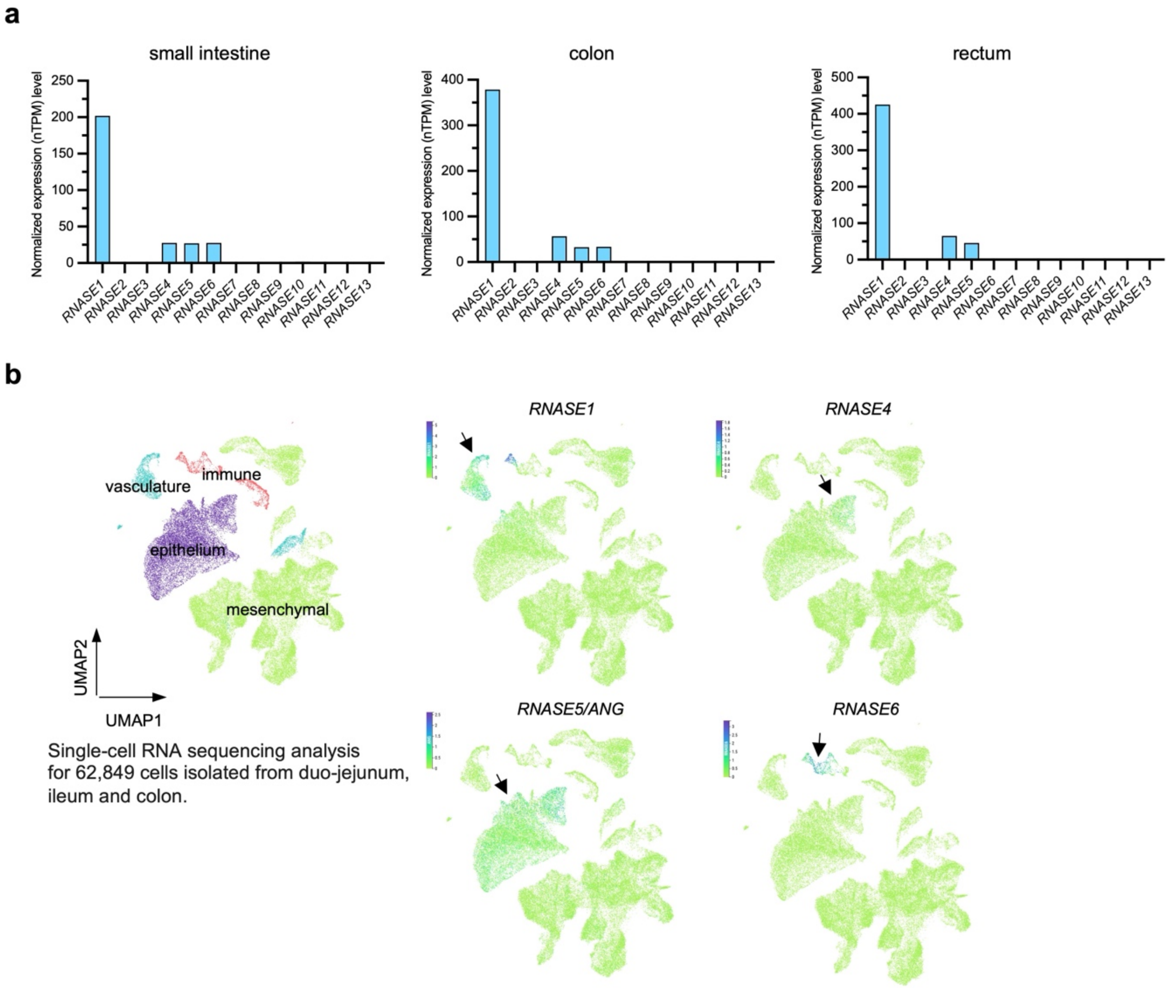

Supplementary Figure 1. Expression profile of *RNASEA* superfamily members in the intestine.
a Expression levels of the *RNASEA* superfamily members (*RNASE1-13*) in the small intestine, colon, and rectum based on Human Protein Atlas (HPA) and Genotype-Tissue Expression (GTEx) transcriptomics datasets, sourced from www.proteinatlas.org. **b** *RNASE1*, *4*, *5*, and *6* expression levels in various intestinal cell types based on single-cell RNA sequencing analysis data from Gut Cell Survey, sourced from www.gutcellatlas.org. The black arrow highlights the cell cluster wherein the indicated *RNASEA* superfamily member is mainly expressed.

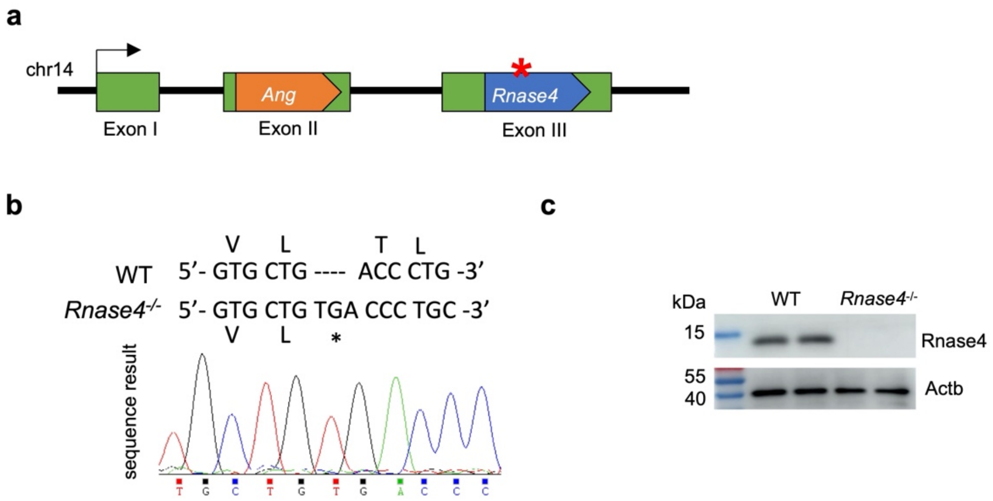

Supplementary Figure 2. Generation of *Rnase4* knockout mice.

a *Rnase4* gene structure and target site (red asterisk) for transcription activator-like effector nuclease-based knockout strategy. **b** Confirmation of successful insertion of TG in the *Rnase4* gene by DNA sequencing (c.49_50insTG, resulting in premature translation termination), leading to the generation of *Rnase4*$^{-/-}$ mice. **c** Rnase4 protein levels in the colons of WT and *Rnase4*$^{-/-}$ mice by immunoblotting.

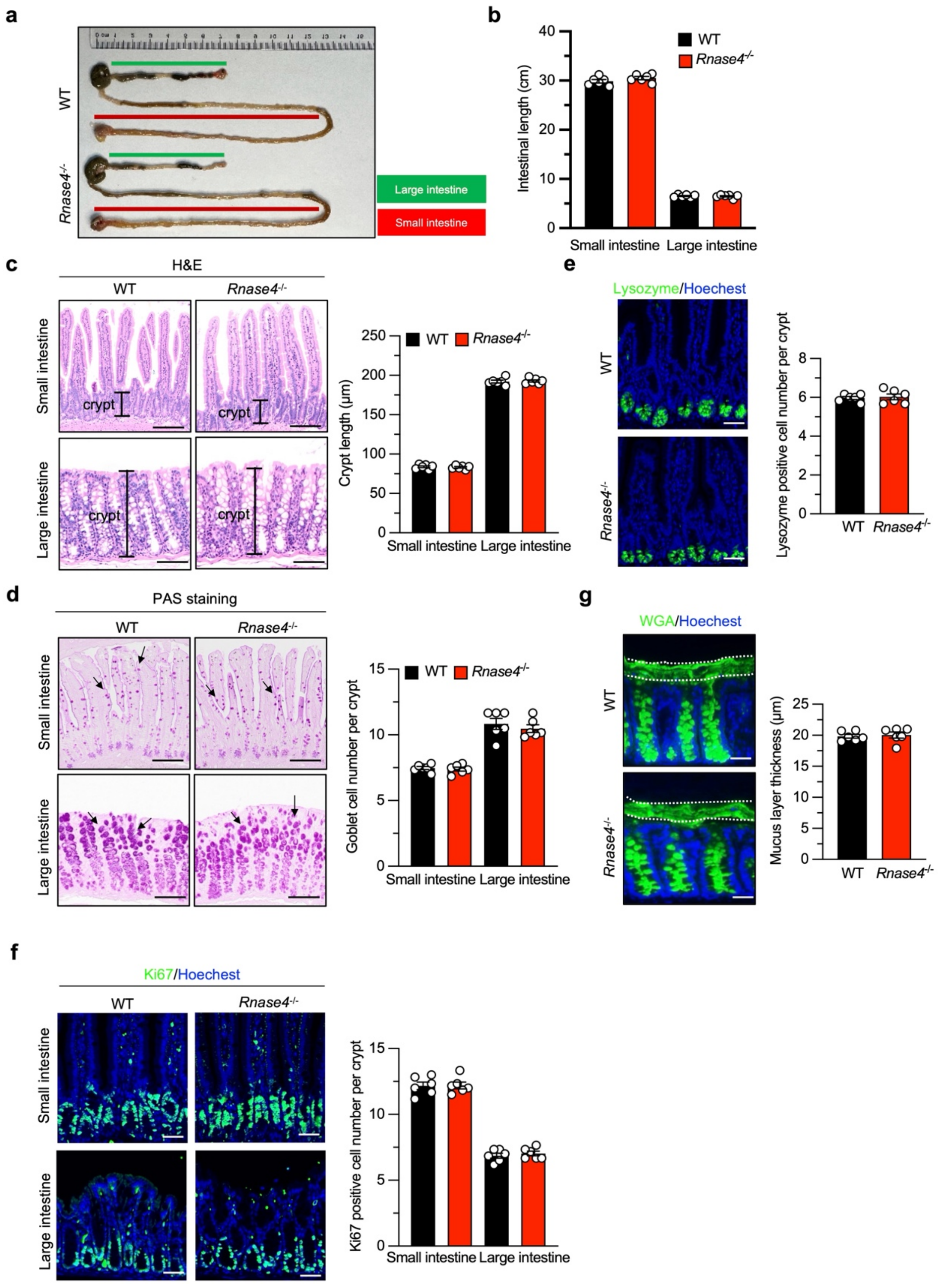

Supplementary Figure 3. Phenotypic characterization of intestines from WT and *Rnase4*$^{-/-}$ mice.
a Gross morphology of the intestines from 2-month-old mice. **b** Quantitative data of the total intestinal length (n=6). **c-g** Representative staining images and corresponding quantitative data of crypt length (**c**), number of goblet cells per crypt (**d**), number of lysozyme positive cells per crypt (**e**), number of Ki67 positive cells per crypt (**f**), and mucus layer thickness (**g**) in the intestines (n=6). Scale bar, 75 μm in H&E and PAS staining; 25 μm in immunofluorescence staining. Data are presented as mean ± SEM. Statistical significance are determined by two-tailed unpaired Student's *t*-test (**b**, **c**, **d**, **e**, **f**, and **g**).

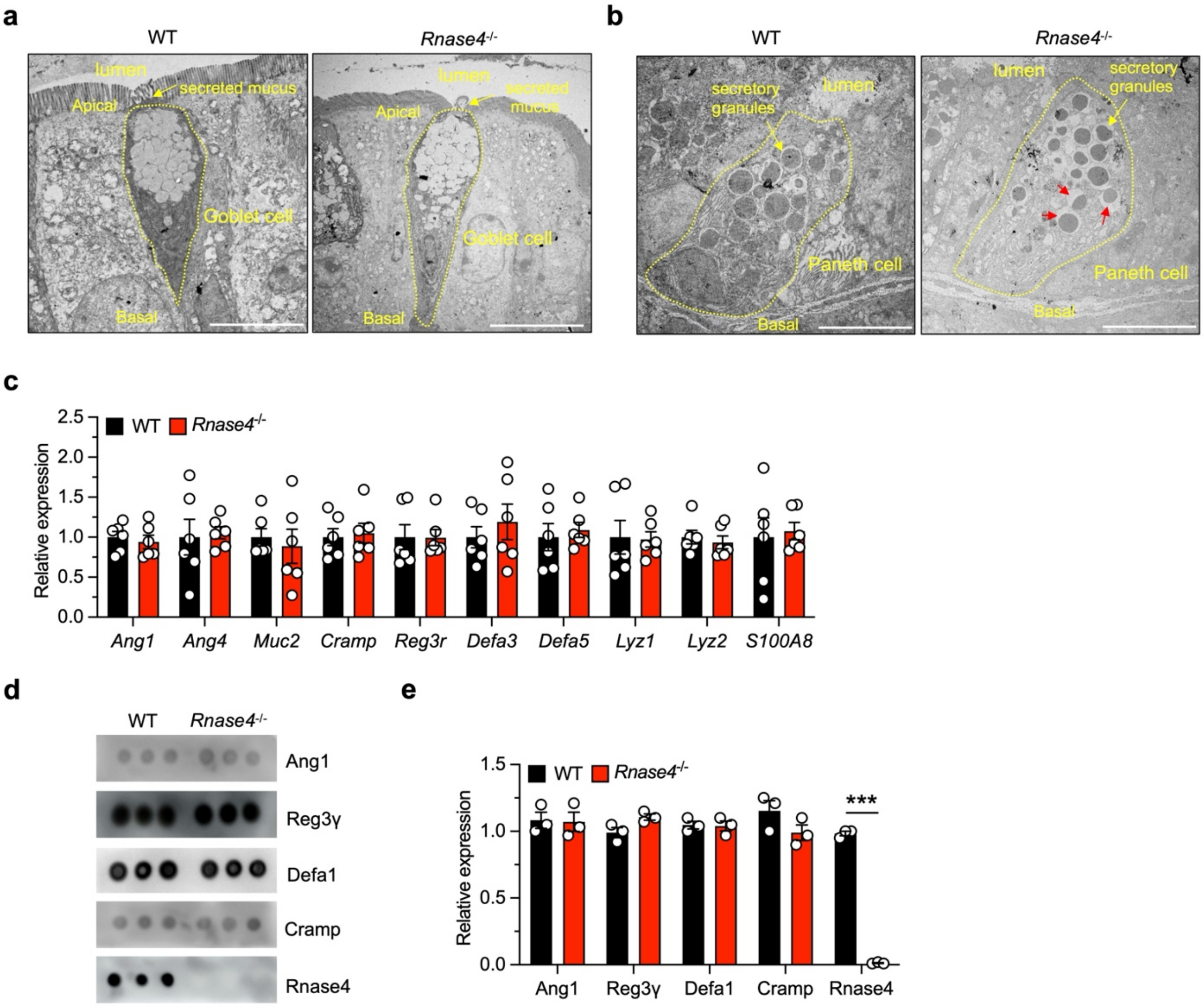

Supplementary Figure 4. The goblet and Paneth cell morphology and expression of key antimicrobial genes in intestines from WT and *Rnase4*$^{-/-}$ mice.

a, b Representative transmission electron microscopy images of goblet and Paneth cells. Red arrows indicate vacuoles within the secretory granules. Scale bar, 5 μm. **c** Quantitative mRNA expression of the selected antimicrobial genes in the colons, measured by quantitative PCR (n=6 mice). **d, e** Immunoblots and their quantification for key antimicrobial proteins in stool samples (n=3 mice). Data are presented as mean ± SEM, *** $p<0.001$ by two-tailed unpaired Student's *t*-test (**c** and **e**).

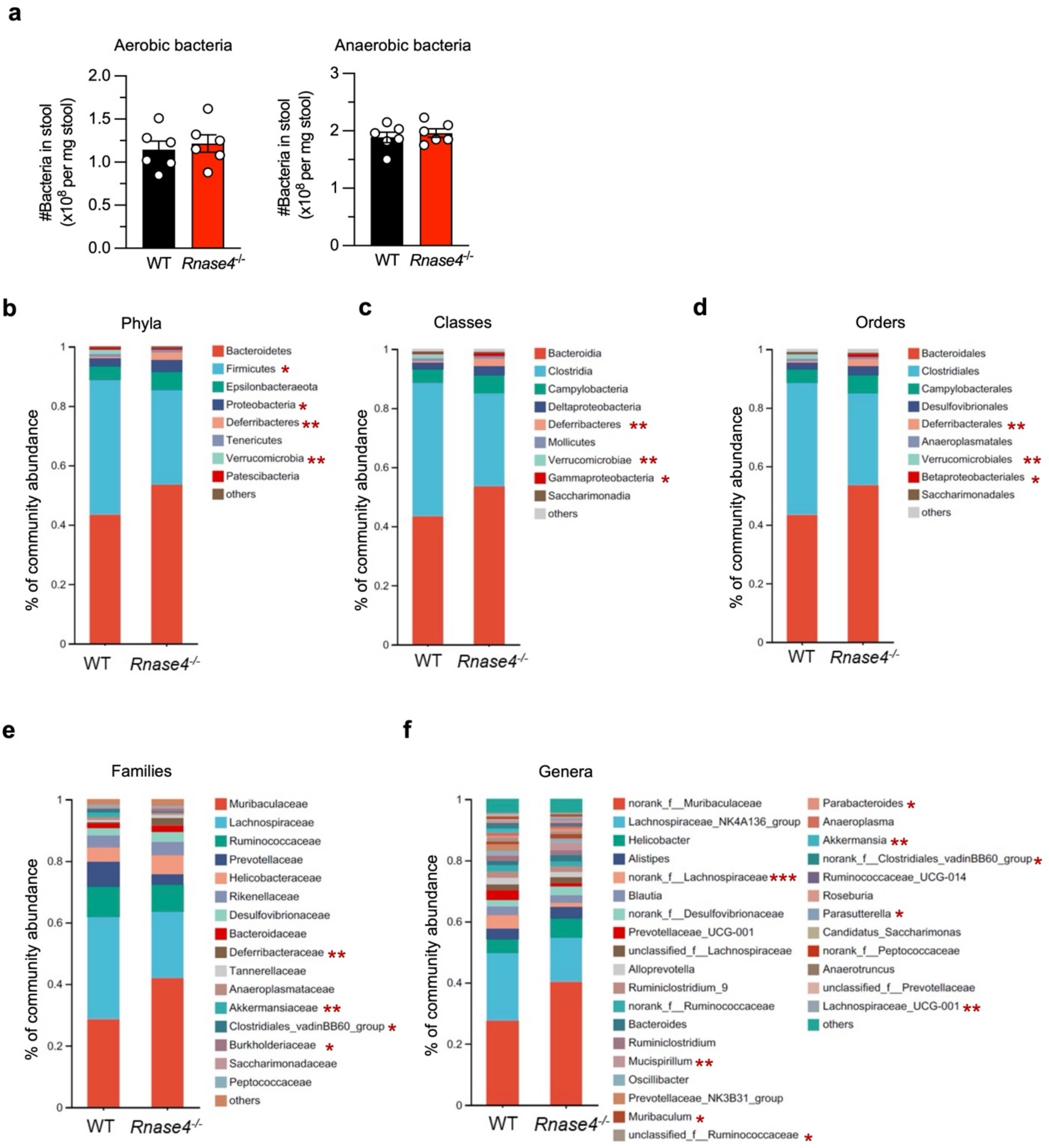

Supplementary Figure 5. Bacterial load and composition in WT and *Rnase4*$^{-/-}$ mice.

a Bacterial load in stool samples from WT and *Rnase4*$^{-/-}$ mice (n=6). **b-f** Relative abundance of bacterial phyla (**b**), classes (**c**), orders (**d**), families (**e**), and genera (**f**) in stool samples from WT and *Rnase4*$^{-/-}$ mice (n=6). Data are presented as mean ± SEM. * p<0.05; ** p<0.01; *** p<0.001 by two-tailed unpaired Student's *t*-test (**b**, **c**, **d**, **e** and **f**).

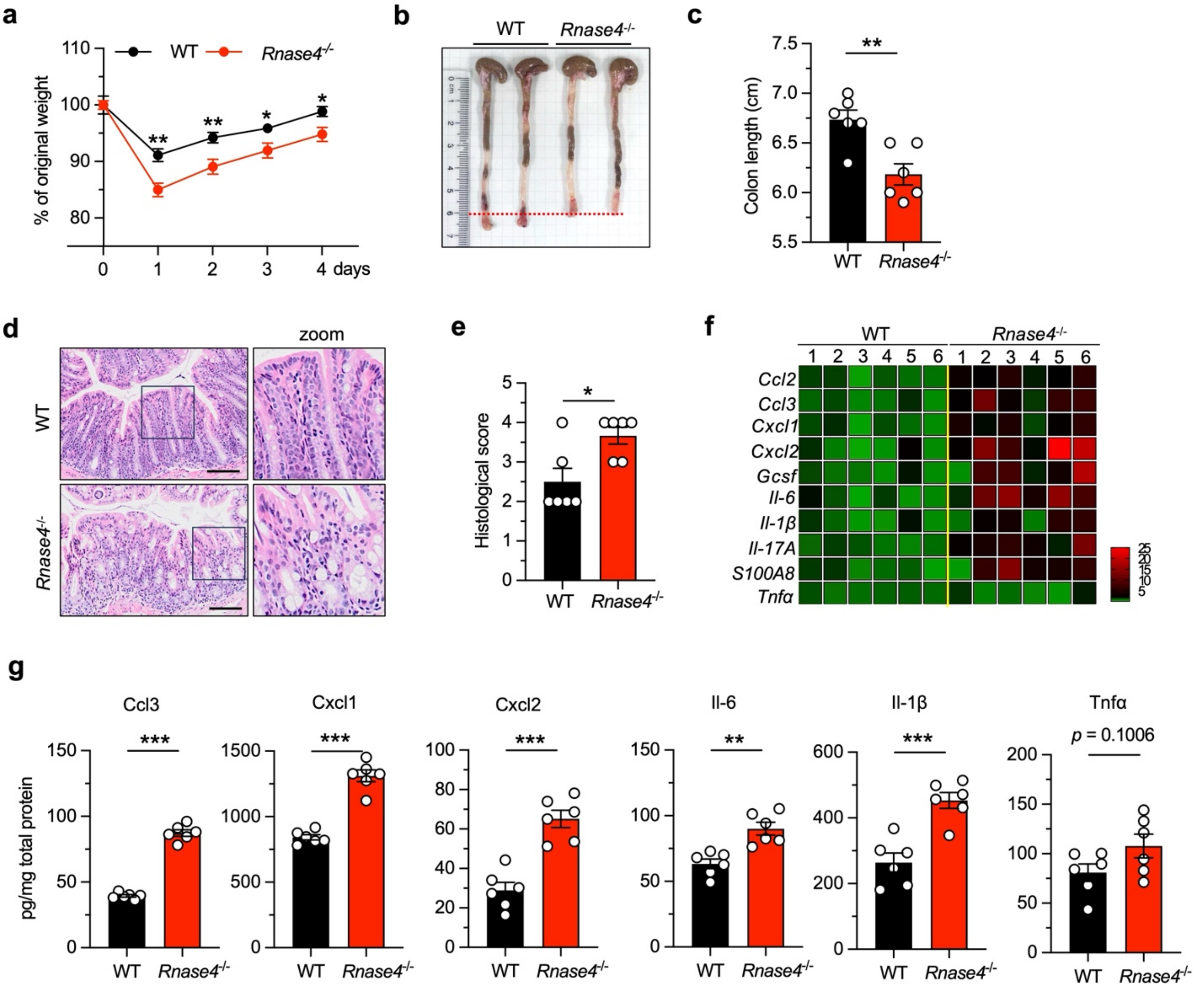

Supplementary Figure 6. Effect of *Rnase4* deficiency on TNBS-induced colitis.

a Body weight loss of WT and *Rnase4*$^{-/-}$ mice during TNBS treatment (n=6). **b** Representative pictures of the colons from WT and *Rnase4*$^{-/-}$ mice at the end of TNBS treatment. **c** Colon length of WT and *Rnase4*$^{-/-}$ mice after TNBS treatment (n=6). **d, e** Representative H&E staining image (**d**) and histological score of colonic sections (**e**) from WT and *Rnase4*$^{-/-}$ mice after TNBS treatment (n=6). **f, g** Quantitative mRNA expression (**f**) and protein level (**g**) of indicated cytokines in the colons of WT and *Rnase4*$^{-/-}$ mice after TNBS treatment, measured by quantitative PCR (n=6). Scale bar, 50 μm. Data are presented as mean ± SEM (**a**, **c**, **e** and **g**) and as mean for (**f**); * $p<0.05$; ** $p<0.01$; *** $p<0.001$ by two-tailed unpaired Student's *t*-test (**a**, **c** and **g**) or two-tailed Mann–Whitney U test (**e**).

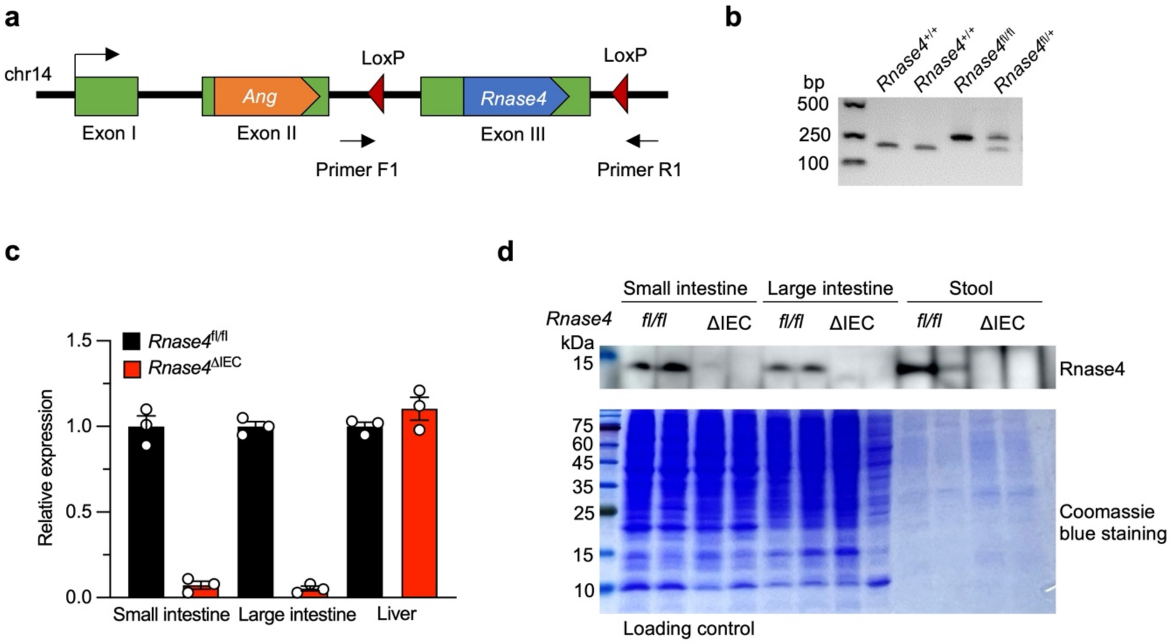

Supplementary Figure 7. Generation and Validation of *Rnase4*$^{\Delta IEC}$ Mice.

a *Rnase4* gene structure and target site (red triangle) for the CRISPR/Cas9 conditional knockout strategy. **b** Genotyping of *Rnase4*$^{+/+}$, *Rnase4*$^{fl/+}$, and *Rnase4*$^{fl/fl}$ mice by PCR. **c** Quantitative mRNA expression of *Rnase4* in the colons of *Rnase4*$^{fl/fl}$ and *Rnase4*$^{\Delta IEC}$ mice, measured by quantitative PCR (n=3). *Rnase4* expression in the liver served as a control. **d** RNASE4 protein level in small intestine, large intestine, and stool sample of *Rnase4*$^{fl/fl}$ and *Rnase4*$^{\Delta IEC}$ mice by immunoblotting. Coomassie blue staining shows the total protein loading amount. Data are presented as mean ± SEM (**c**).

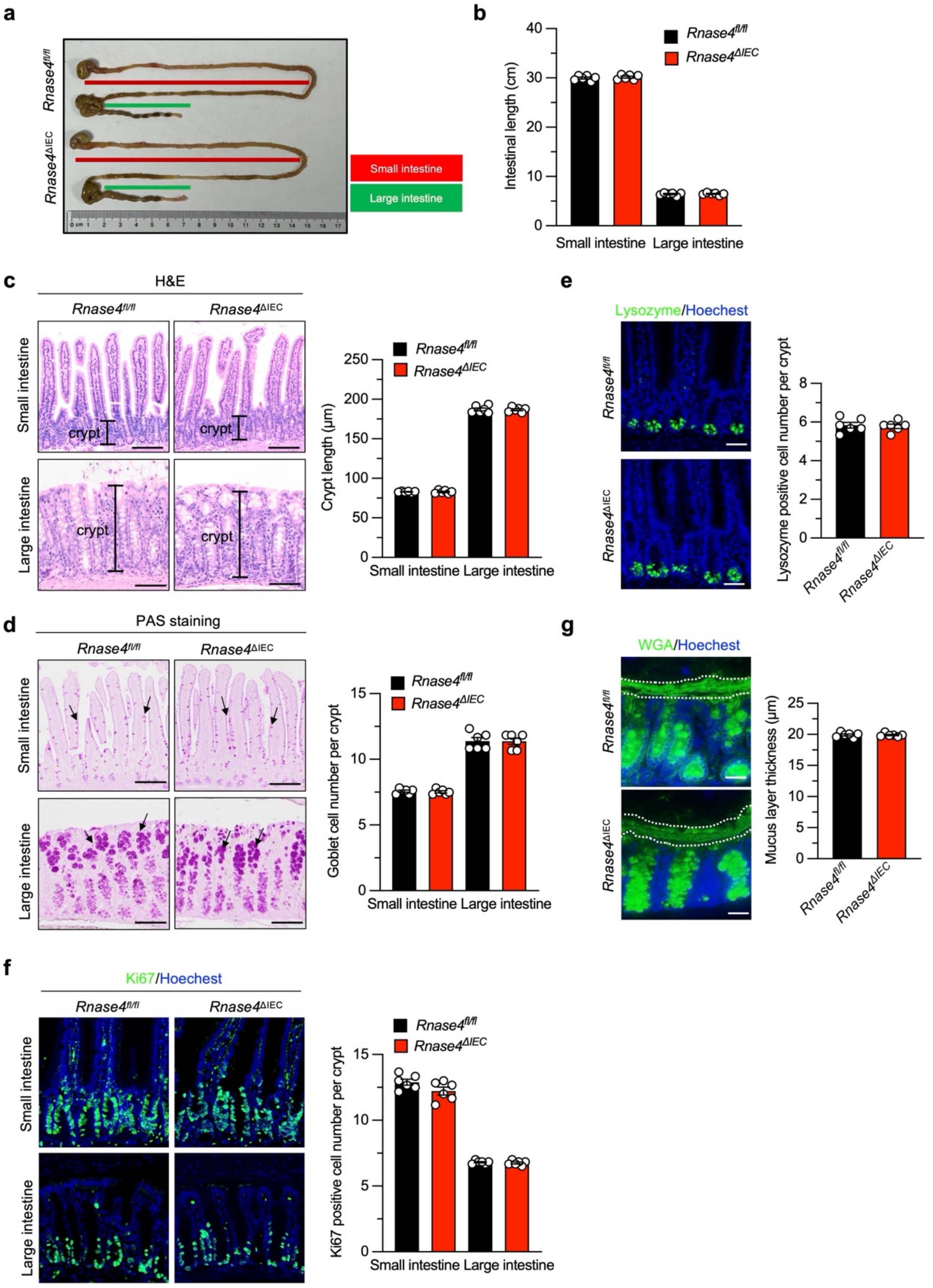

Supplementary Figure 8. Phenotypic characterization of intestines from *Rnase4*$^{fl/fl}$ and *Rnase4*$^{\Delta IEC}$ mice.
a Gross morphology of the intestines from 2-month-old mice. **b** Quantitative data of the total intestinal length (n=6). **c-g** Representative staining images and corresponding quantitative data of crypt length (**c**), number of goblet cells per crypt (**d**), number of lysozyme positive cells per crypt (**e**), number of Ki67 positive cells per crypt (**f**), and mucus layer thickness (**g**) in the intestines (n=6). Scale bar, 75 μm in H&E and PAS staining; 25 μm in immunofluorescence staining. Data are presented as mean ± SEM. Statistical significance are determined by two-tailed unpaired Student's *t*-test (**b**, **c**, **d**, **e**, **f** and **g**).

a

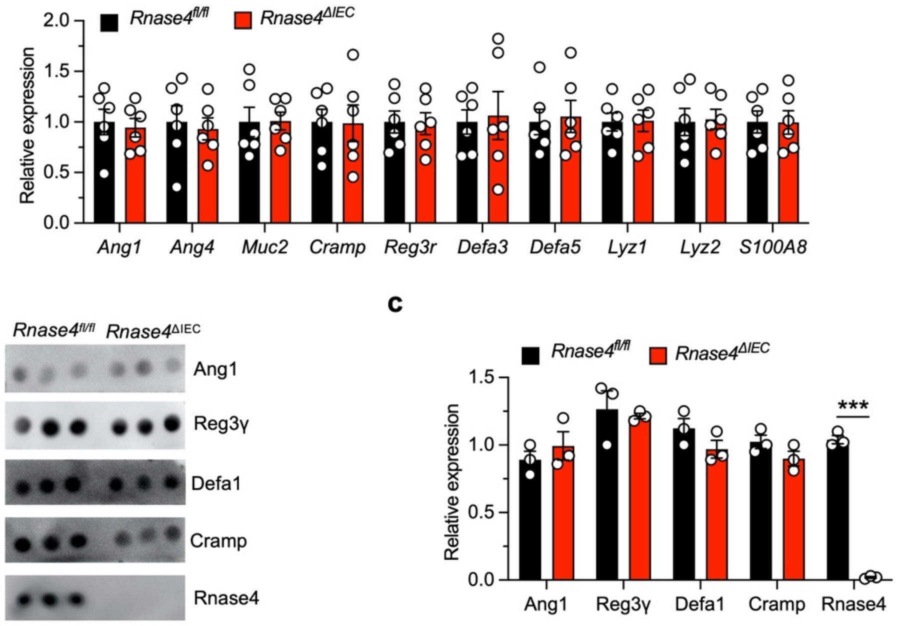

Supplementary Figure 9. The expression of key antimicrobial genes in intestines from *Rnase4$^{fl/fl}$* and *Rnase4*$^{\Delta IEC}$ mice.

a Quantitative mRNA expression of the selected antimicrobial genes in the colons, measured by quantitative PCR (n=6). **b-c** Immunoblots and their quantification for key antimicrobial proteins in stool samples (n=3). Data are presented as mean ± SEM, *** p<0.001 by two-tailed unpaired Student's t-test (**a** and **c**).

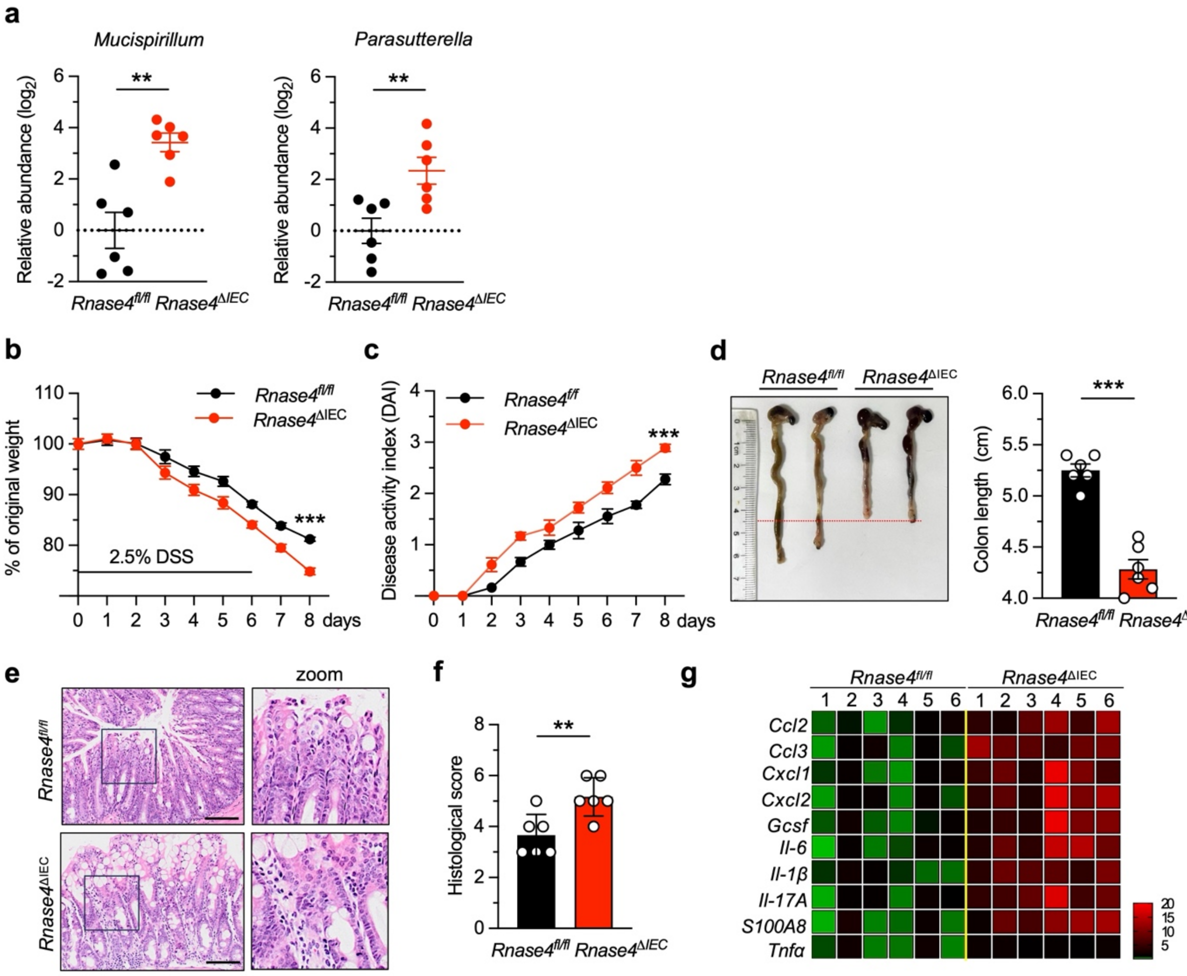

Supplementary Figure 10. *Rnase4*$^{\Delta IEC}$ mice are more sensitive to DSS-induced colitis.

a Abundance of *Mucispirillum* and *Parasutterella* in the gut microbiota of *Rnase4*$^{fl/fl}$ and *Rnase4*$^{\Delta IEC}$ mice, detected by quantitative PCR analysis (n=6). **b-c** Body weight loss (**b**) and disease activity index (**c**) of *Rnase4*$^{fl/fl}$ and *Rnase4*$^{\Delta IEC}$ mice during 2.5% DSS treatment (n=6). **d-f** Colon length (**d**), representative H&E staining image (**e**) and histological score of colonic section (**f**) from *Rnase4*$^{fl/fl}$ and *Rnase4*$^{\Delta IEC}$ mice on day 8 after 2.5% DSS administration (n=6). **g** Quantitative mRNA expression of indicated cytokines in colons from *Rnase4*$^{fl/fl}$ and *Rnase4*$^{\Delta IEC}$ mice on day 8 after 2.5% DSS administration (n=6). Scale bar, 50 μm in H&E staining. Data are presented as mean ± SEM for (**a**, **b**, **c**, **d**, and **f**) and as mean for (**g**); ** p<0.01; *** p<0.001 by two-tailed unpaired Student's t-test (**a**, **b**, **d** and **f**) or two-tailed Mann–Whitney U test (**c**).

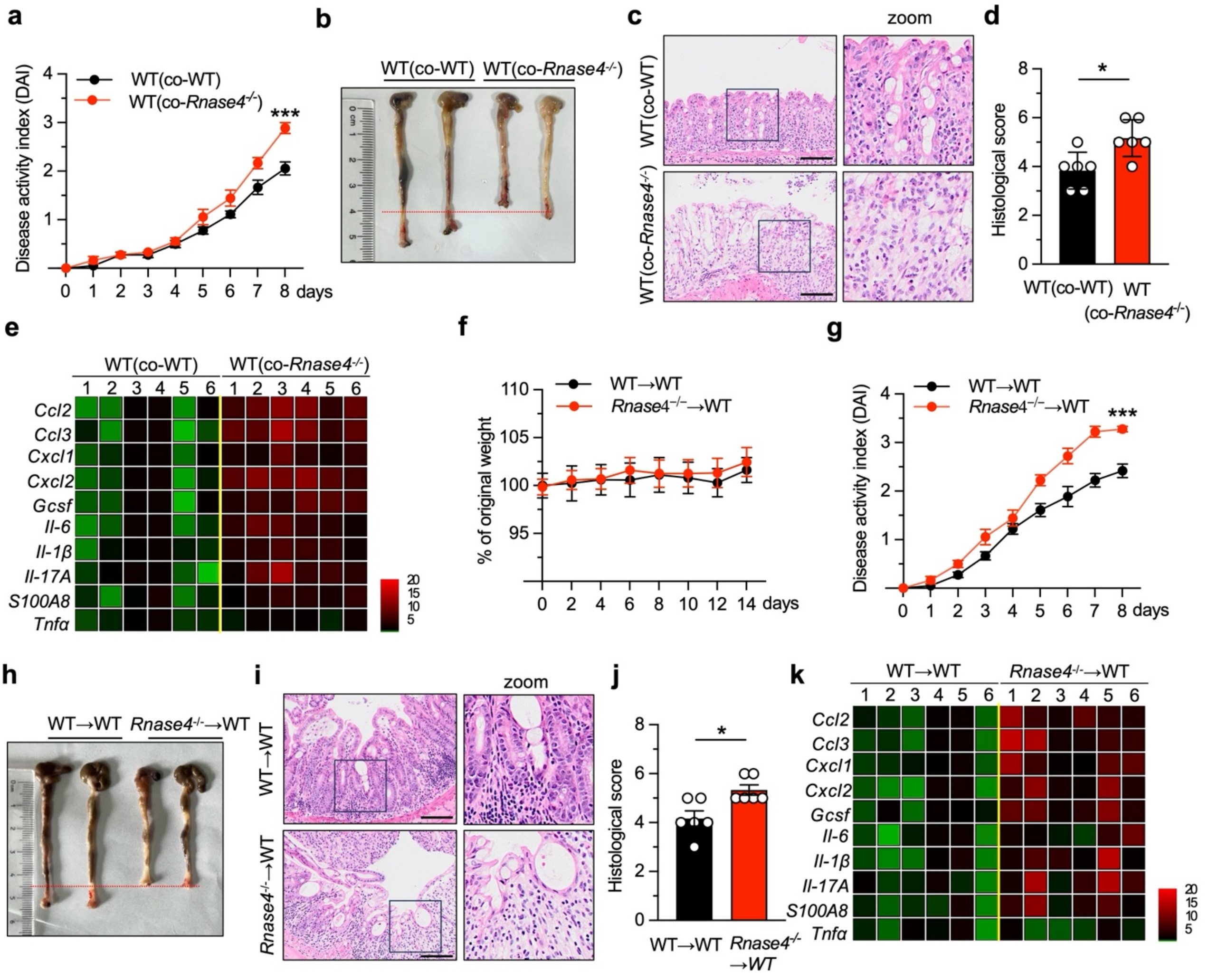

Supplementary Figure 11. Rnase4-regulated bacteria are associated with colitis.

a-e Disease activity index (**a**), representative colon image (**b**), representative H&E staining image (**c**), histological score of colonic section (**d**), and quantitative mRNA expression of the indicated cytokines (**e**) of WT (co-WT) and WT (co-*Rnase4*$^{-/-}$) mice with DSS-induced colitis (n=6). **f** Body weight change of WT→WT and *Rnase4*$^{-/-}$→WT mice during fecal microbiota transplantation. **g-k** Disease activity index (**g**), representative colon image (**h**), representative H&E staining image (**i**), histological score of colonic section (**j**), and quantitative mRNA expression of the indicated cytokines (**k**) of WT→WT and *Rnase4*$^{-/-}$→WT mice with DSS-induced colitis (n=6). Scale bar, 50 μm in H&E staining. Data are presented as mean ± SEM for (**a**, **d**, **f**, **g** and **j**) and as mean for (**e and k**); * p<0.05; *** p<0.001 by two-tailed unpaired Student's t-test (**d** and **g**) or two-tailed Mann–Whitney test (**a** and **j**).

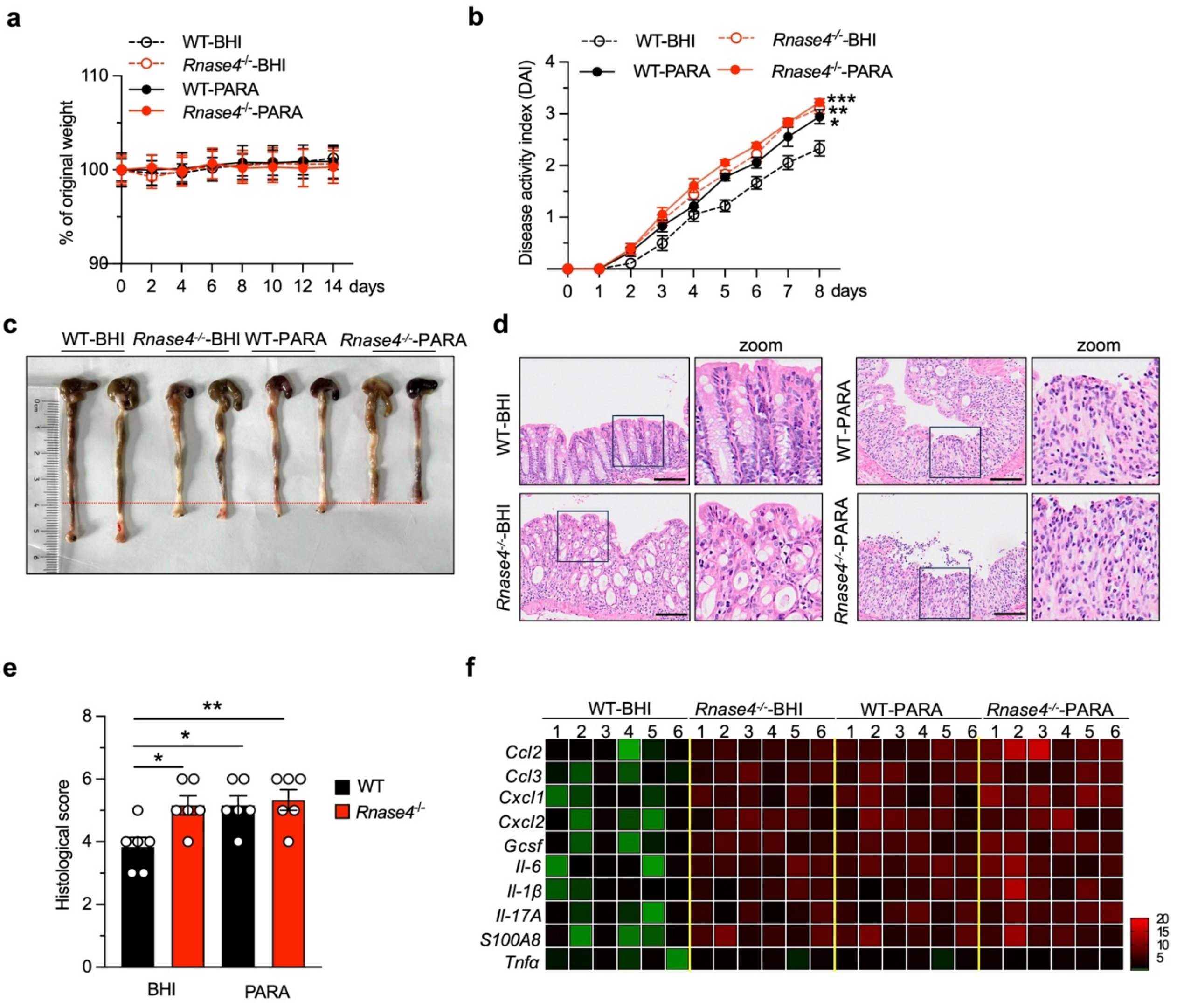

Supplementary Figure 12. Over-colonization of *Parasutterella* exacerbates colitis in mice.

a Body weight change in mice gavaged with *Parasutterella* strain or vehicle control. **b-f** Disease activity index (**b**), representative colon image (**c**), representative H&E staining image (**d**), histological score of colonic section (**e**) and quantitative mRNA expression of the indicated cytokines (**f**) of WT-BHI, WT-PARA, *Rnase4*$^{-/-}$-BHI and *Rnase4*$^{-/-}$-PARA mice during DSS-induced colitis (n=6). Scale bar, 50 μm in H&E staining. Data are presented as mean ± SEM for (**a**, **b** and **e**) and as mean for (**f**); * $p<0.05$; ** $p<0.01$; *** $p<0.001$ by two-tailed unpaired Student's *t*-test (**a** and **e**) or two-tailed Mann–Whitney U test (**b**)

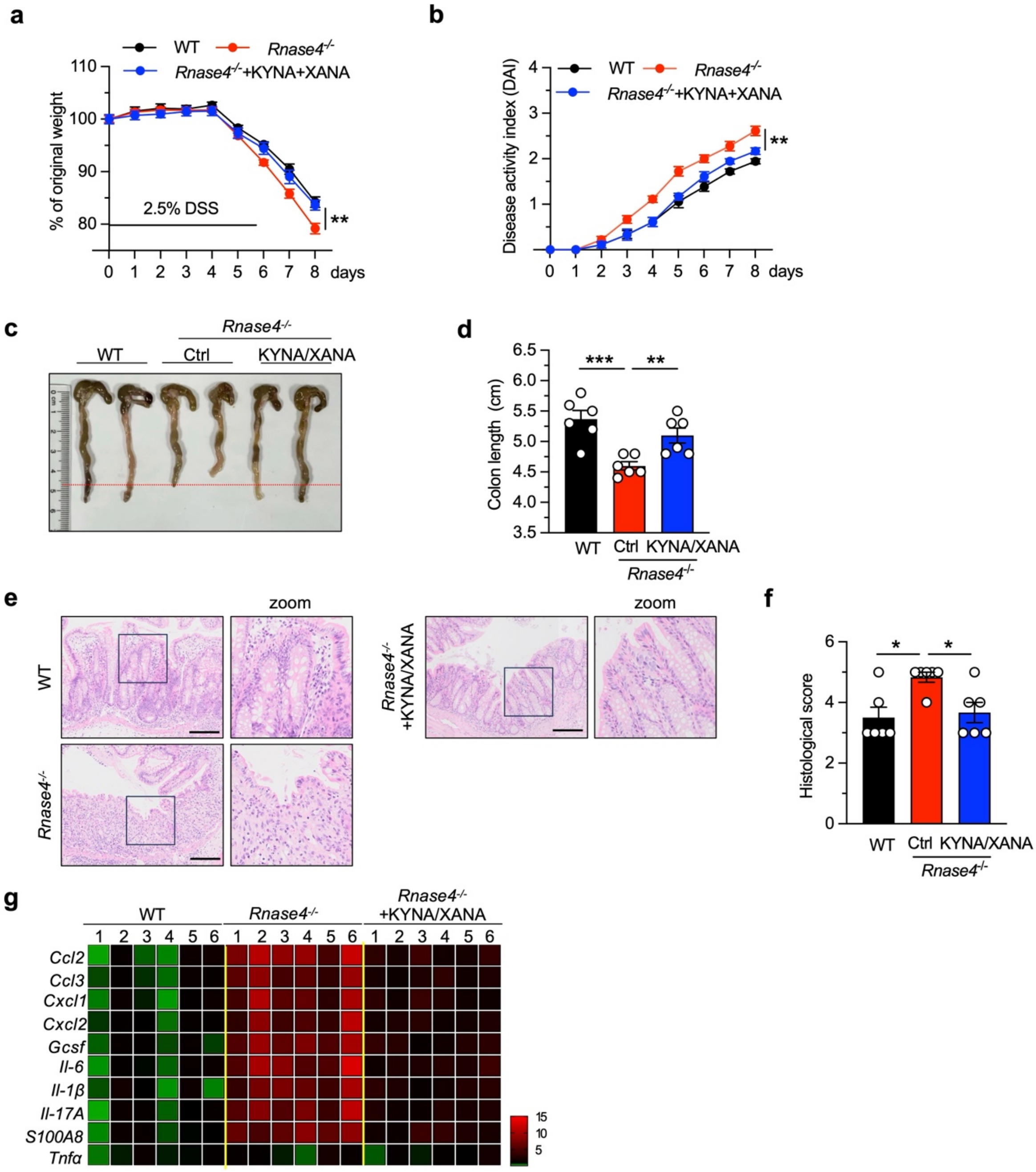

Supplementary Figure 13. KYNA and XANA treatment suppresses DSS-induced colitis in *Rnase4*$^{-/-}$ mice

a-g Body weight loss (**a**), Disease activity index (**b**), representative colon image (**c**), colon length (**d**), representative H&E staining image (**e**), histological score of colonic section (**f**) and quantitative mRNA expression of the indicated cytokines (**g**) of WT, *Rnase4*$^{-/-}$ and *Rnase4*$^{-/-}$ mice supplemented with KYNA and XANA during DSS-induced colitis (n=6). Scale bar, 50 μm in H&E staining. Data are presented as mean ± SEM for (**a**, **b**, **d** and **f**) and as mean for (**g**); * p<0.05; ** p<0.01; *** p<0.001by two-tailed unpaired Student's t-test (**a** and **d**) or two-tailed Mann–Whitney U test (**b** and **f**)

a

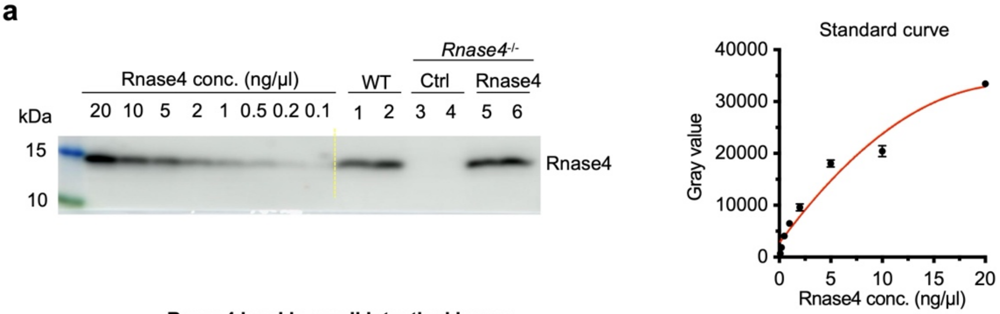

Rnase4 level in small intestinal lumen

No.	Gray value	Rnase4 conc. (ng/μl)	Rnase4 conc. in lumen (μM, 14.7 KDa)	Rnase4 conc. in lumen (μg/g total protein)
#1	19104.39	7.22	0.49	0.36
#2	20364.20	7.91	0.53	0.40
#5	22108.91	8.95	0.61	0.45
#6	22259.51	9.25	0.63	0.46

b

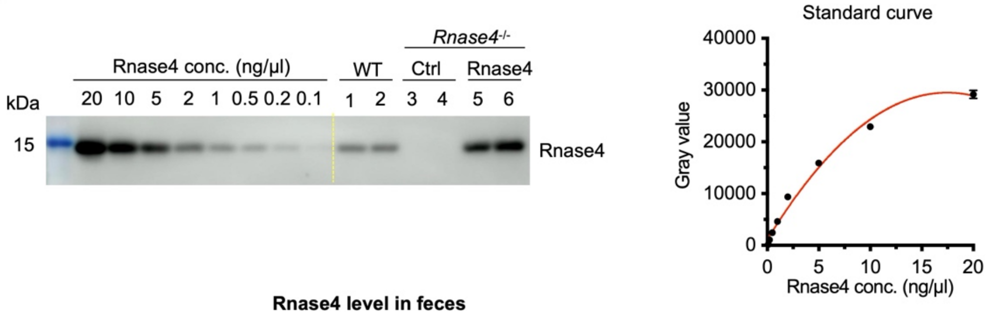

Rnase4 level in feces

No.	Gray value	Rnase4 conc. (ng/μl)	Rnase4 conc. in feces (μM, 14.7 KDa)	Rnase4 conc. in feces (μg/g total protein)
#1	5664.60	1.47	0.10	0.07
#2	6876.07	1.86	0.12	0.09
#5	15219.37	5.06	0.34	0.25
#6	21188.74	8.01	0.54	0.40

Supplementary Figure 14. Rnase4 level in the small intestinal lumen and feces of mice during Rnase4 administration.

a-b Immunoblotting analysis of Rnase4 protein level in the small intestinal lumen or feces from WT mice and *Rnase4*$^{-/-}$ mice treated with or without Rnase4. The standard curve on the right shows the linear relationship between Rnase4 concentration and gray value. Table showing the gray values, Rnase4 concentrations (ng/μl), Rnase4 concentrations in the lumen or feces (μM, based on Rnase4 molecular weight of 14.7 kDa), and Rnase4 concentrations in the lumen (μg/g total protein) for each sample.

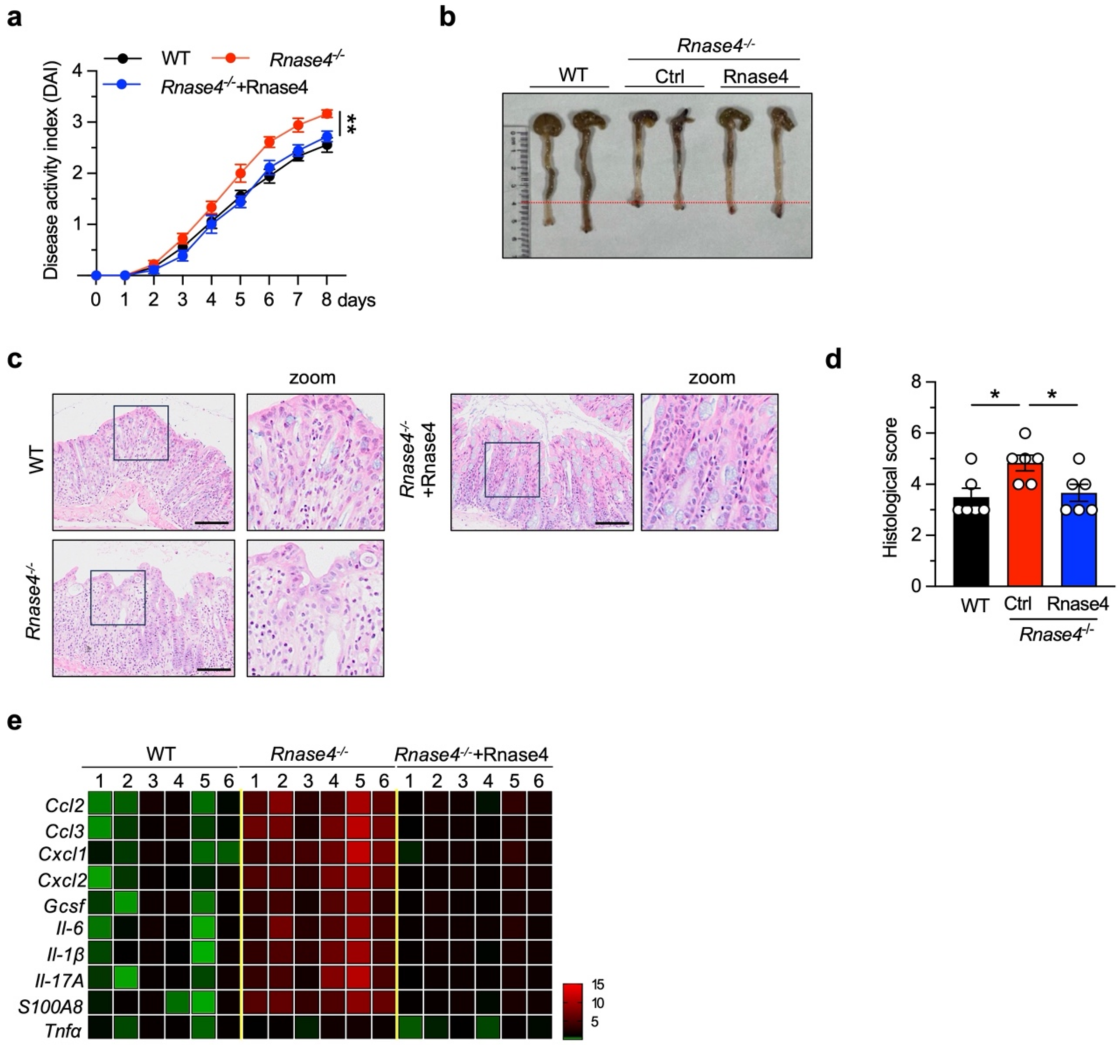

Supplementary Figure 15. Exogenous Rnase4 suppresses DSS-induced colitis in *Rnase4*$^{-/-}$ mice.

a-e Disease activity index (**a**), representative colon image (**b**), representative H&E staining image (**c**), histological score of colonic section (**d**) and quantitative mRNA expression of the indicated cytokines (**e**) of WT, *Rnase4*$^{-/-}$, and *Rnase4*$^{-/-}$+Rnase4 mice in response to DSS treatment (n=6). Scale bar, 50 µm in H&E staining. Data are presented as mean ± SEM for (**b** and **e**) and as mean for (**e**); * p<0.05; ** p<0.01 by two-tailed unpaired Student's t-test (**a**) or two-tailed Mann–Whitney U test (**d**)

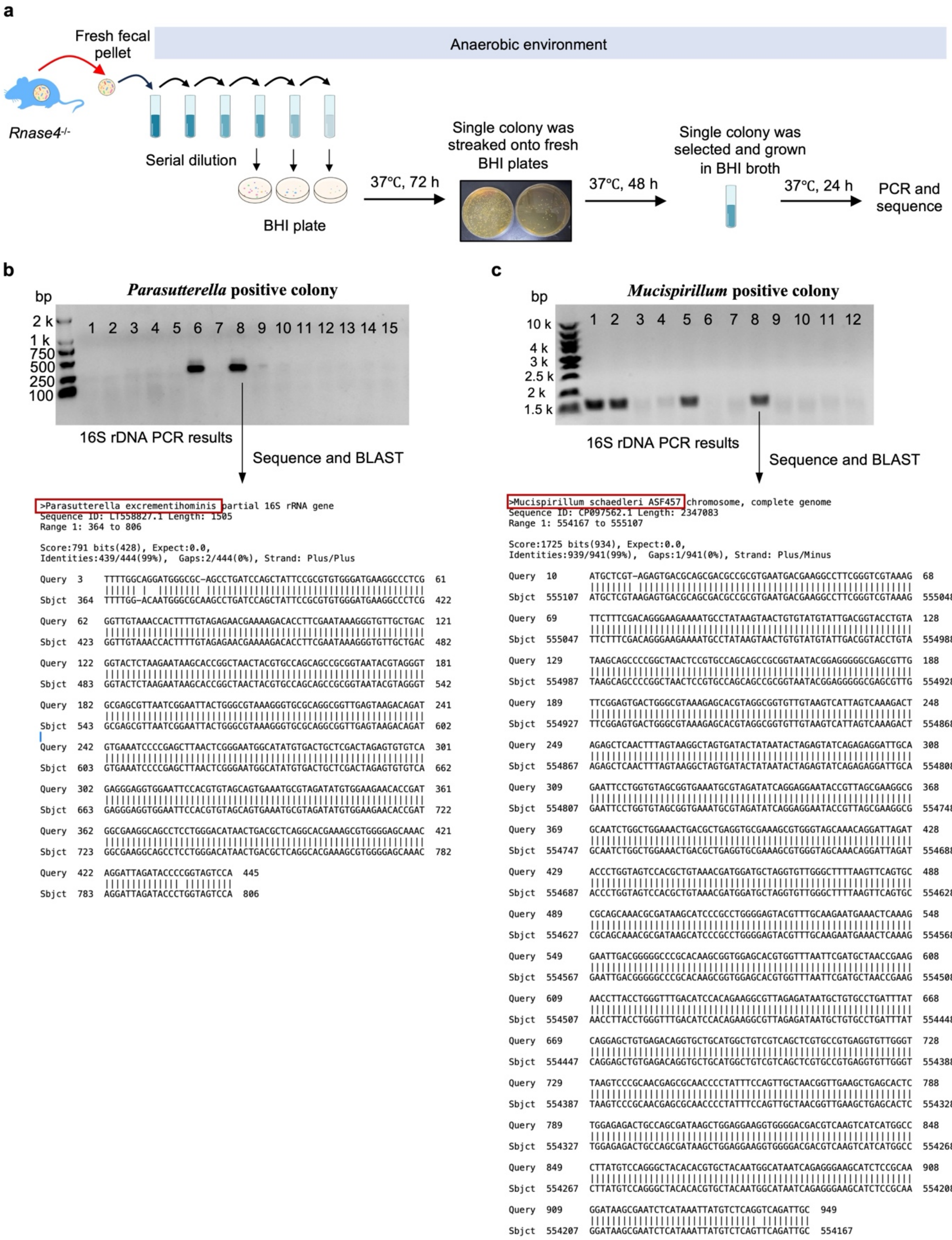

Supplementary Figure 16. Bacterial strain isolation and identification procedure.

a Workflow for isolating and identifying bacterial strains from the fecal samples of *Rnase4*$^{-/-}$ mice. **b-c** Images of agarose gel electrophoresis show the 16S rDNA PCR results for *Parasutterella* (**b**) or *Mucispirillum* (**c**) positive colony. The PCR amplicon is sequenced, and a BLAST search is performed to identify the bacterial strain.

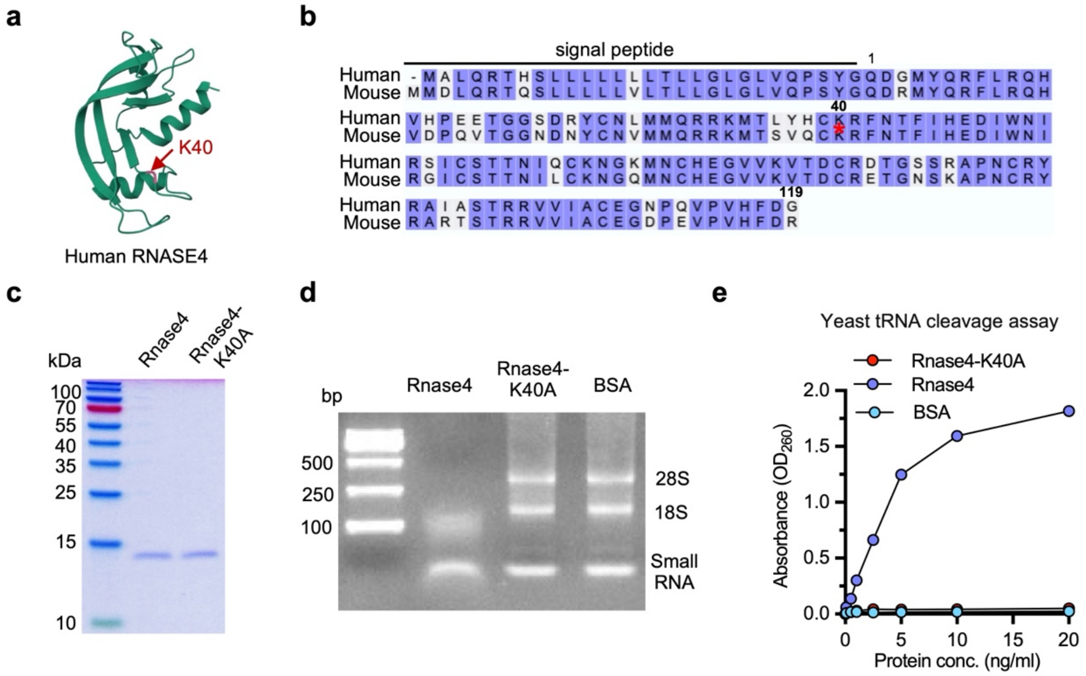

Supplementary Figure 17. Characterization of Rnase4 protein.

a Crystal structure of the ribonuclease enzyme RNASE4 (PDB ID: 1RNF). **b** Alignment of Rnase4 protein sequences from human and mouse, revealing a high degree of conservation. **c** SDS-PAGE analysis of purified recombinant Rnase4 protein, showing a single band at the expected molecular weight. **d** Agarose gel demonstrating the ribonucleolytic activity of recombinant Rnase4 and its variant Rnase4-K40A on HeLa cell total RNA. **e** Yeast tRNA cleavage assay comparing the ribonucleolytic activities of recombinant Rnase4, Rnase4-K40A, and BSA at increasing protein concentrations (n=3 independent experiments). The dose-dependent increase in absorbance at 260 nm (A260) for Rnase4 demonstrates their ribonucleolytic activity, while the lack of significant change in A260 for Rnase4-K40I and BSA control confirms the absence of ribonucleolytic activity. Data are presented as mean ± SEM (**e**).

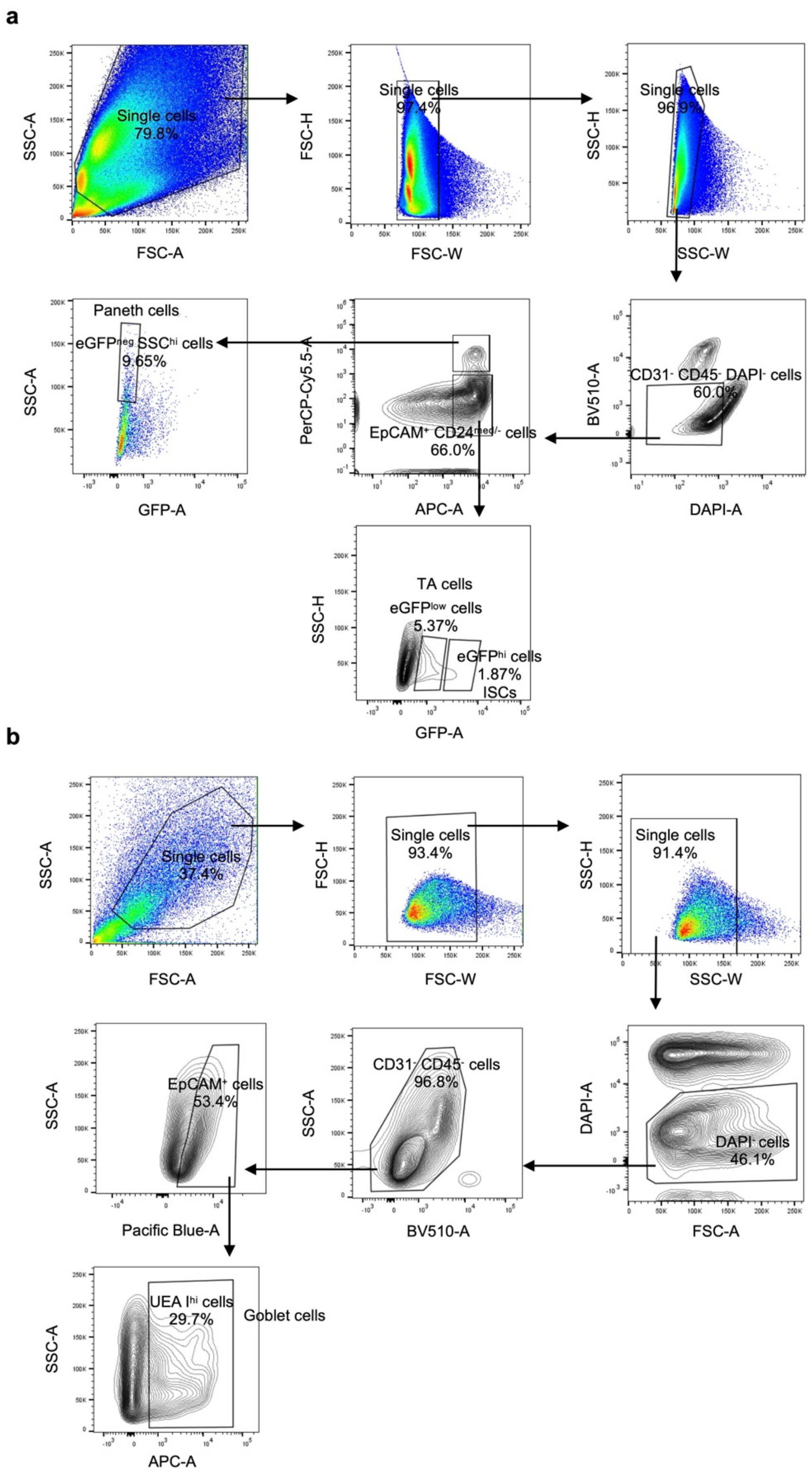

Supplementary Figure 18. Gating strategy of flow cytometry

a Representative gating strategy of flow cytometry for sorting intestinal stem cells (ISCs), transit-amplifying (TA) cells, and Paneth cells from mouse intestinal crypts. **b** Representative gating strategy of flow cytometry for sorting colonic goblet cells. The gating panels correspond to Figure 1c.

Supplementary Tables

Supplementary Table 1. Bacteria Strains

Strain	Classification (Phylum; Class; Order; Family; Genus)
Parasutterella excrementihominis	Pseudomonadota; Betaproteobacteria; Burkholderiales; Sutterellaceae; Parasutterella
Mucispirillum schaedleri	Deferribacterota; Deferribacteres; Deferribacterales; Deferribacteraceae; Mucispirillum

Supplementary Table 2. Basic Information of IBD Cohort

Cohort 1

Characteristics	Ctrl (n=25)	IBD (n=73)	
		UC (n=27)	CD (n=46)
Age, Year (mean ± SD)	42.24 ± 13.11	38.44 ± 12.85	36.52 ± 13.64
Male / Female n (%)	16 (64.0%) / 9 (36.0%)	20 (74.1%) / 7 (25.9%)	32 (69.6%)/14 (30.4%)
Age at diagnosis, Year (mean ± SD)	NA	31.26 ± 10.32	35.39 ± 16.03
**Montreal classification**			
A1 / A2 / A3 (n)	NA	NA	3 / 36 / 7
L1 / L2 / L3 / L4 (n)	NA	NA	0 / 14 / 32 / 0
B1 / B2 / B3 (n)	NA	NA	8 / 28 / 10
E1 / E2 / E3 (n)	NA	2 / 1 / 24	NA
HBI (median, IQR)	NA	NA	9 (6-16)
PMS (median, IQR)	NA	6 (3-8)	NA

Cohort 2

Characteristics	Ctrl (n=45)	IBD (n=64)	
		UC (n=15)	CD (n=49)
Age, Year (mean ± SD)	39.1 ± 13.4	37.0 ± 9.9	35.9 ± 10.3
Male / Female n (%)	26 (58%) / 19 (42%)	8 (53%) / 7 (47%)	34 (69%)/15 (31%)
Age at diagnosis, Year (mean ± SD)	NA	28.3 ± 11.0	30.9 ± 13.6
**Montreal classification**			
A1 / A2 / A3 (n)	NA	NA	3 / 39 / 7
L1 / L2 / L3 / L4 (n)	NA	NA	0 / 17 / 32 / 0
B1 / B2 / B3 (n)	NA	NA	8 / 29 / 12
E1 / E2 / E3 (n)	NA	2 / 1 / 12	NA
HBI (median, IQR)	NA	NA	8 (6-16)
PMS (median, IQR)	NA	5 (3-8)	NA

Ctrl: healthy participants. IBD: inflammatory bowel disease. UC: ulcerative colitis. CD: Crohn's disease. NA: not-available. Montreal classification of extent of UC: E1, ulcerative proctitis; E2, left sided UC; E3, extensive UC. Age at diagnosis by Montreal classification for CD: A1, <16; A2, 16-40; A3, >40. Localization of disease by Montreal classification: L1, ileal; L2, colonic; L3: ileocolonic; L4: upper gastrointestinal tract. Disease behavior for Montreal classification for CD: B1, non-stricturing; B2, stricturing; B3, penetrating. HBI, Harvey-Bradshaw Index; PMS, partial Mayo score; IQR, interquartile range.

Supplementary Table 3. Genotyping Primers

Name	Forward primer (5′→3′)	Reverse primer (5′→3′)
Rnase4	CAGAGCCCAGTCCTTACCATCCTT	AGCGTTTGCACTGGACAGAAGTCA
Rnase4$^{fl/fl}$	TAATTCTGACTTGTCTGGGTTGT	GAAGAAGTCGTGCTGCTTCATGT
Villin-cre	GTGTTTGGTTTGGTTTCCTCTGCATAAGA	GCAGGCAAATTTTGGTGTACGGTCA

Supplementary Table 4. Primers for Bacterial Analysis

Name	Forward primer (5′→3′)	Reverse primer (5′→3′)
Universal bacterial	ACTCCTACGGGAGGCAGCAGT	ATTACCGCGGCTGCTGGC
Parasutterella	AACGTRTCCGCTCGTGGGGGAC	CGGAATAGCTGGATCAGGCTTG
M.schaedleri	AGTTTCATTCTTGCAAACGT	GGCCATTACCCTACCAACTA

Supplementary Table 5. Probes Used in FISH

Probe	Fluorophore Sequence (5'-3')
Para-cy3	AACGTRTCCGCTCGTGGGGGAC
Muc-cy5	CAGTCACTCCGAACAACGCT

Supplementary Table 6. RT-qPCR Primers

Gene	species	Forward primer (5′→3′)	Reverse primer (5′→3′)
Actb	mouse	GTGACGTTGACATCCGTAAAGA	GCCGGACTCATCGTACTCC
Il-1β	mouse	GAAATGCCACCTTTTGACAGTG	TGGATGCTCTCATCAGGACAG
Il-6	mouse	TCTATACCACTTCACAAGTCGGA	GAATTGCCATTGCACAACTCTTT
Il-17a	mouse	GGCCCTCAGACTACCTCAAC	TCTCGACCCTGAAAGTGAAGG
S100a8	mouse	AAATCACCATGCCCTCTACAAG	CCCACTTTTATCACCATCGCAA
Tnfα	mouse	CTGAACTTCGGGGTGATCGG	GGCTTGTCACTCGAATTTTGAGA
Cxcl1	mouse	ACTGCACCCAAACCGAAGTC	TGGGGACACCTTTTAGCATCTT
Cxcl2	mouse	CCAACCACCAGGCTACAGG	GCGTCACACTCAAGCTCTG
Ccl2	mouse	AACTCTCACTGAAGCCAGCTCT	CGTTAACTGCATCTGGCTGA
Ccl3	mouse	TGTACCATGACACTCTGCAAC	CAACGATGAATTGGCGTGGAA
Gcsf	mouse	ATGGCTCAACTTTCTGCCCAG	CTGACAGTGACCAGGGGAAC
Ang1	mouse	CATCCCAACAGGAAGGAAGGA	ACCTGGAGTCATCCTGAGCC
Ang4	mouse	GGTTGTGATTCCTCCAACTCTG	CTGAAGTTTTCTCCATAAGGGCT
Muc2	mouse	AGGGCTCGGAACTCCAGAAA	CCAGGGAATCGGTAGACATCG
Cramp	mouse	GGCTGTGGCGGTCACTATC	GTCTAGGGACTGCTGGTTGAA
Reg3γ	mouse	TTCCTGTCCTCCATGATCAAA	CATCCACCTCTGTTGGGTTC
Defa3	mouse	ATCTGGTATGCTATTGTAGAAA	GTGGCCTCAGTACTCATGT
Defa5	mouse	TCAAAAAAGCTGATATGCTATTG	AGCTGCAGCAGAATACGAAAG
Lay1	mouse	GAGACCGAAGCACCGACTATG	CGGTTTTGACATTGTGTTCGC
Lay2	mouse	ATGGAATGGCTGGCTACTATGG	ACCAGTATCGGCTATTGATCTGA
Tph1	mouse	AGTTGCGGTATGACCTTGAT	AGGCGAGAGACATTGCTAA
Ido1	mouse	GTACATCACCATGGCGTATG	CGAGGAAGAAGCCCTTGTC
IDO1	human	GGTCATGGAGATGTCCGTAA	ACCAATAGAGAGACCAGGAAGAA
Kmo	mouse	ATGGCATCGTCTGATACTCAGG	AGCTTCGTACACATCAACTTGAA
Kynu	mouse	CCTTCGCCTCTTGAGCTTCC	AGAGCAACCCTCTCATCTGTT
Kyat3	mouse	TTCAAAAACGCCAAACGAATCG	GATGACCAAAGCCTCTTGTGT
Kyat4	mouse	GCGTTACCGAAGCCTTCAAGA	GGAGCACGTAAGGTTTTCCGT

Supplementary Table 7. Antibody information

Name	Catalogue numbers	Company	Dilutions
CD31-PE	#102407	BioLegend	1:1000
CD45-PE	#103105	BioLegend	1:1000
EpCAM-APC	#17-5791	Thermo Fisher Scientific	1:1000
CD24-PerCP-Cyanine 5.5	#562360	BD Biosciences	1:1000
CD31-BV 510	#563454	BD Biosciences	1:1000
CD45-BV 510	#563204	BD Biosciences	1:1000
EpCAM-eFluorTM 450	#48-5791-82	Thermo Fisher Scientific	1:1000
UEA I-DyLight 649	#DL-1068-1	Vector Laboratories	1:1000
mouse anti-Ki67	#550609	BD Biosciences	1:400
rabbit anti-Lysozyme	#AB108508	Abcam	1:400
rabbit anti-Rnase4	NA	Homemade	1:100
donkey anti-rabbit IgG conjugated to Alexa Fluor 488	A21206	Thermo Fisher Scientific	1:1000
donkey anti-rabbit IgG conjugated to Alexa Fluor 555	A31572	Thermo Fisher Scientific	1:1000
anti-mouse IgG conjugated to Alexa Fluor 488	A28175	Thermo Fisher Scientific	1:1000
mouse anti-ACTB antibody	#60008-1-Ig	Proteintech	1:2000
rabbit anti-IDO1 antibody	#13268-1-AP	Proteintech	1:1000
mouse anti-GAPDH antibody	#60004-1-Ig	Proteintech	1:2000
rabbit anti-Ang1 antibody	NA	Homemade	1:1000
rabbit anti-Reg3γ antibody	#PS03255	Abmart	1:2000
abbit anti-Defa1anitbody	#PA3475	Abmart	1:2000
rabbit anti-Cramp antibody	#TD6523	Abmart	1:2000
Goat anti-mouse IgG HRP-conjugated secondary antibody	#31430	Thermo Fisher Scientific	1:1000
Goat anti-rabbit IgG HRP-conjugated secondary antibody	#31460	Thermo Fisher Scientific	1:1000
