# Supplementary material for: Ribonuclease 4 functions as an intestinal antimicrobial protein to maintain gut microbiota and metabolite homeostasis
Source: Nat Commun. 2024 Jul 10;15:5778. doi: 10.1038/s41467-024-50223-1 (PMC11237007; doi:10.1038/s41467-024-50223-1)
Supplement: Supplementary file 3 — Reporting Summary [file 41467_2024_50223_MOESM3_ESM.pdf]

nature portfolio

Corresponding author(s): Jinghao Sheng

Last updated by author(s): Jun 25, 2024

Reporting Summary

Nature Portfolio wishes to improve the reproducibility of the work that we publish. This form provides structure for consistency and transparency in reporting. For further information on Nature Portfolio policies, see our Editorial Policies and the Editorial Policy Checklist.

Statistics

For all statistical analyses, confirm that the following items are present in the figure legend, table legend, main text, or Methods section.

n/a	Confirmed	
☐	☒	The exact sample size (*n*) for each experimental group/condition, given as a discrete number and unit of measurement
☐	☒	A statement on whether measurements were taken from distinct samples or whether the same sample was measured repeatedly
☐	☒	The statistical test(s) used AND whether they are one- or two-sided<br>*Only common tests should be described solely by name; describe more complex techniques in the Methods section.*
☒	☐	A description of all covariates tested
☐	☒	A description of any assumptions or corrections, such as tests of normality and adjustment for multiple comparisons
☐	☒	A full description of the statistical parameters including central tendency (e.g. means) or other basic estimates (e.g. regression coefficient) AND variation (e.g. standard deviation) or associated estimates of uncertainty (e.g. confidence intervals)
☐	☒	For null hypothesis testing, the test statistic (e.g. *F*, *t*, *r*) with confidence intervals, effect sizes, degrees of freedom and *P* value noted<br>*Give P values as exact values whenever suitable.*
☒	☐	For Bayesian analysis, information on the choice of priors and Markov chain Monte Carlo settings
☒	☐	For hierarchical and complex designs, identification of the appropriate level for tests and full reporting of outcomes
☒	☐	Estimates of effect sizes (e.g. Cohen's *d*, Pearson's *r*), indicating how they were calculated

Our web collection on statistics for biologists contains articles on many of the points above.

Software and code

Policy information about availability of computer code

Data collection	NA
Data analysis	All statistical analyses were performed using GraphPad Prism 10.

For manuscripts utilizing custom algorithms or software that are central to the research but not yet described in published literature, software must be made available to editors and reviewers. We strongly encourage code deposition in a community repository (e.g. GitHub). See the Nature Portfolio guidelines for submitting code & software for further information.

Data

Policy information about availability of data

All manuscripts must include a data availability statement. This statement should provide the following information, where applicable:

- Accession codes, unique identifiers, or web links for publicly available datasets
- A description of any restrictions on data availability
- For clinical datasets or third party data, please ensure that the statement adheres to our policy

There are no restrictions on data availability in the current work. The raw 16s rDNA sequencing data have been deposited in NCBI's Sequence Read Archive (SRA) under BioProject accession number PRJNA1008371 (https://www.ncbi.nlm.nih.gov/bioproject/?term=PRJNA1008371). The Single cell sequencing results based on Human Protein Atlas (HPA) and The Genotype-Tissue Expression (GTEx) transcriptomics datasets, sourced from www.proteinatlas.org.

Research involving human participants, their data, or biological material

Policy information about studies with human participants or human data. See also policy information about sex, gender (identity/presentation), and sexual orientation and race, ethnicity and racism.

Reporting on sex and gender	N/A
Reporting on race, ethnicity, or other socially relevant groupings	N/A
Population characteristics	N/A
Recruitment	The human samples of IBD patients and healthy participants were collected from the biological sample bank in Inflammatory Bowel Disease Centre, Sir Run Run Shaw Hospital affiliated to ZJU School of Medicine.
Ethics oversight	The use of tissue samples complied with the relevant requirements of the Health Commission of the People's Republic of China and the Medical Ethics Committee of Zhejiang University School of Medicine.

Note that full information on the approval of the study protocol must also be provided in the manuscript.

Field-specific reporting

Please select the one below that is the best fit for your research. If you are not sure, read the appropriate sections before making your selection.

[x] Life sciences [ ] Behavioural & social sciences [ ] Ecological, evolutionary & environmental sciences

For a reference copy of the document with all sections, see nature.com/documents/nr-reporting-summary-flat.pdf

Life sciences study design

All studies must disclose on these points even when the disclosure is negative.

Sample size	Each group contained at least 3 samples. For DSS-induced coltis and TNBS-induced colitis model used in the study, 6 mice per group were used for each experiment, in accordance with statistical tests to be performed. Please see methods and figure legends for details.
Data exclusions	No data was excluded from the data analysis.
Replication	Experiments were repeated at least 3 independent experiments with similar results. All experiments were reproduced to reliably support conclusions stated in the manuscript.
Randomization	All samples /organisms were randomly allocated into experimental groups.
Blinding	The investigators were blinded to group allocation during data collection and analysis.

Reporting for specific materials, systems and methods

We require information from authors about some types of materials, experimental systems and methods used in many studies. Here, indicate whether each material, system or method listed is relevant to your study. If you are not sure if a list item applies to your research, read the appropriate section before selecting a response.

Materials & experimental systems

n/a	Involved in the study	
[ ]	[x]	Antibodies
[ ]	[x]	Eukaryotic cell lines
[x]	[ ]	Palaeontology and archaeology
[ ]	[x]	Animals and other organisms
[x]	[ ]	Clinical data
[x]	[ ]	Dual use research of concern
[x]	[ ]	Plants

Methods

n/a	Involved in the study	
[x]	[ ]	ChIP-seq
[ ]	[x]	Flow cytometry
[x]	[ ]	MRI-based neuroimaging

Antibodies

Antibodies used	CD31-PE (#102407; BioLegend)<br>CD45-PE (#103105; BioLegend)

EpCAM-APC (#17-5791; Thermo Fisher Scientific)
CD24-PerCP-Cyanine 5.5 (#562360; BD Biosciences)
CD31-BV 510 (#563454; BD Biosciences)
CD45-BV 510 (#563204; BD Biosciences)
EpCAM-eFluorTM 450 (#48-5791-82; Thermo Fisher Scientific)
UEA I-DyLight 649 (#DL-1068-1; Vector Laboratories)
mouse anti-Ki67 (#550609; BD Biosciences)
rabbit anti-Lysozyme (#AB108508; Abcam)
rabbit anti-Rnase4 (NA; Homemade)
donkey anti-rabbit IgG conjugated to Alexa Fluor 488 (A21206; Thermo Fisher Scientific)
donkey anti-rabbit IgG conjugated to Alexa Fluor 555 (A31572; Thermo Fisher Scientific)
anti-mouse IgG conjugated to Alexa Fluor 488 (A28175; Thermo Fisher Scientific)
mouse anti-ACTB antibody (#60008-1-Ig; Proteintech)
rabbit anti-IDO1 antibody (#13268-1-AP; Proteintech)
mouse anti-GAPDH antibody (#60004-1-Ig; Proteintech)
rabbit anti-Ang1 antibody (NA; Homemade)
rabbit anti-Reg3γ antibody (#PS03255; Abmart)
abbit anti-Defa1 antibody (#PA3475; Abmart)
rabbit anti-Cramp antibody (#TD6523; Abmart)
Goat anti-mouse IgG HRP-conjugated secondary antibody (#31430; Thermo Fisher Scientific)
Goat anti-rabbit IgG HRP-conjugated secondary antibody (#31460; Thermo Fisher Scientific)

Validation

All antibodies were obtained from reputable vendors. Refer to their websites for validation data and relevant citations for the species and application used in this study.

Eukaryotic cell lines

Policy information about cell lines and Sex and Gender in Research

Cell line source(s)	HICE6 Purchased from American Type Culture Collection, cat# CRL-3266.
Authentication	Cell lines were authenticated by Short Tandem Repeat test.
Mycoplasma contamination	No mycoplasma contamination.
Commonly misidentified lines (See ICLAC register)	No commonly misidentified cell lines were used.

Animals and other research organisms

Policy information about studies involving animals; ARRIVE guidelines recommended for reporting animal research, and Sex and Gender in Research

Laboratory animals	All mice are C57BL/6 background. Transcription activator-like effector nuclease (TALEN)-based Rnase4 knockout mice (Rnase4-/-) and CRISPR-Cas9-based Rnase4 conditional knockout mice (Rnase4fl/fl) were generated in collaboration with Cyagen Biosciences. For mouse breeding, Rnase4-/- mice and their wild-type (WT) controls were generated from the same heterozygous Rnase4+/- parents, while Rnase4ΔIEC mice and their controls (Rnase4fl/fl) were generated from Rnase4fl/fl mice and Villin-cre mice . Littermates were randomly assigned to experimental groups. All mice were maintained in specific pathogen-free (SPF) conditions (temperatures of ~ 18-23 °C with 40–60% humidity) with a standard 12-hour daylight cycle at the Laboratory Animal Centre of Zhejiang University, and provided with water and a standard laboratory diet ad libitum, except where otherwise noted.
Wild animals	The study did not involve wild animals.
Reporting on sex	There was no sex bias in the animals used in this study.
Field-collected samples	The study did not involve samples collected from the filed.
Ethics oversight	All the animal experiments were performed strictly in compliance with Zhejiang University Animal Study Committee’s requirements.

Note that full information on the approval of the study protocol must also be provided in the manuscript.

Plants

Seed stocks	*Report on the source of all seed stocks or other plant material used. If applicable, state the seed stock centre and catalogue number. If plant specimens were collected from the field, describe the collection location, date and sampling procedures.*
Novel plant genotypes	*Describe the methods by which all novel plant genotypes were produced. This includes those generated by transgenic approaches, gene editing, chemical/radiation-based mutagenesis and hybridization. For transgenic lines, describe the transformation method, the number of independent lines analyzed and the generation upon which experiments were performed. For gene-edited lines, describe the editor used, the endogenous sequence targeted for editing, the targeting guide RNA sequence (if applicable) and how the editor was applied.*
Authentication	*Describe any authentication procedures for each seed stock used or novel genotype generated. Describe any experiments used to assess the effect of a mutation and, where applicable, how potential secondary effects (e.g. second site T-DNA insertions, mosiacism, off-target gene editing) were examined.*

Flow Cytometry

Plots

Confirm that:

☐ The axis labels state the marker and fluorochrome used (e.g. CD4-FITC).

☐ The axis scales are clearly visible. Include numbers along axes only for bottom left plot of group (a 'group' is an analysis of identical markers).

☐ All plots are contour plots with outliers or pseudocolor plots.

☐ A numerical value for number of cells or percentage (with statistics) is provided.

Methodology

Sample preparation	A single crypt cell suspension was filtered through a 40 µm cell strainer and simultaneously labelled with the following fluorescence-conjugated antibodies in staining buffer (2 mM EDTA (#AM9260G; Thermo Fisher Scientific) and 3% FBS (#10100147; Thermo Fisher Scientific) in PBS): CD31-PE, CD45-PE, EpCAM-APC and CD24-PerCP-Cyanine 5.5. For sorting colonic goblet cells, the cells labeled with the following antibodies: CD31-BV 510, CD45-BV 510, EpCAM-eFluorTM 450 and UEA I-DyLight 649 (the antibody information detailed in Supplementary Table 7). After gently shaking for 30 min on ice, the cells were washed with staining buffer three times, resuspended at a concentration of 5×106 cells/mL in Advanced DMEM/F12 medium (#12634010; Thermo Fisher Scientific) supplemented with 10 µg/mL DAPI (to distinguish live cells from dead/dying cells, #564907; BD Biosciences), and analyzed with an LSR Fortessa flow cytometer.
Instrument	LSRFortessa™; BD FACSAria™ II
Software	BD FACSDiva™
Cell population abundance	intestinal stem cells: 1.87%; transit-amplifying cells: 5.37%; Paneth cells: 9.65%; colonic goblet cells: 29.7%
Gating strategy	The markers for intestinal stem cells were Lgr5-eGFPhi; EpCAM+; CD24med/−; CD31−; CD45−; DAPI−; transit-amplifying cells were Lgr5-eGFPlow; EpCAM+; CD24med/−; CD31−; CD45−; DAPI−; Paneth cells were Lgr5-eGFPneg; EpCAM+; CD24hi; CD31-; CD45−; DAPI−; Side scatterhi; and colonic goblet cells were UEA Ihi; EpCAM+; CD31-; CD45-; DAPI-.

☒ Tick this box to confirm that a figure exemplifying the gating strategy is provided in the Supplementary Information.
